# Supplementary material for: A high-frequency mobility big-data reveals how COVID-19 spread across professions, locations and age groups
Source: PLoS Comput Biol. 2023 Apr 27;19(4):e1011083. doi: 10.1371/journal.pcbi.1011083 (PMC10168568; doi:10.1371/journal.pcbi.1011083)
Supplement: S1 Fig — (PDF) [file pcbi.1011083.s001.pdf]

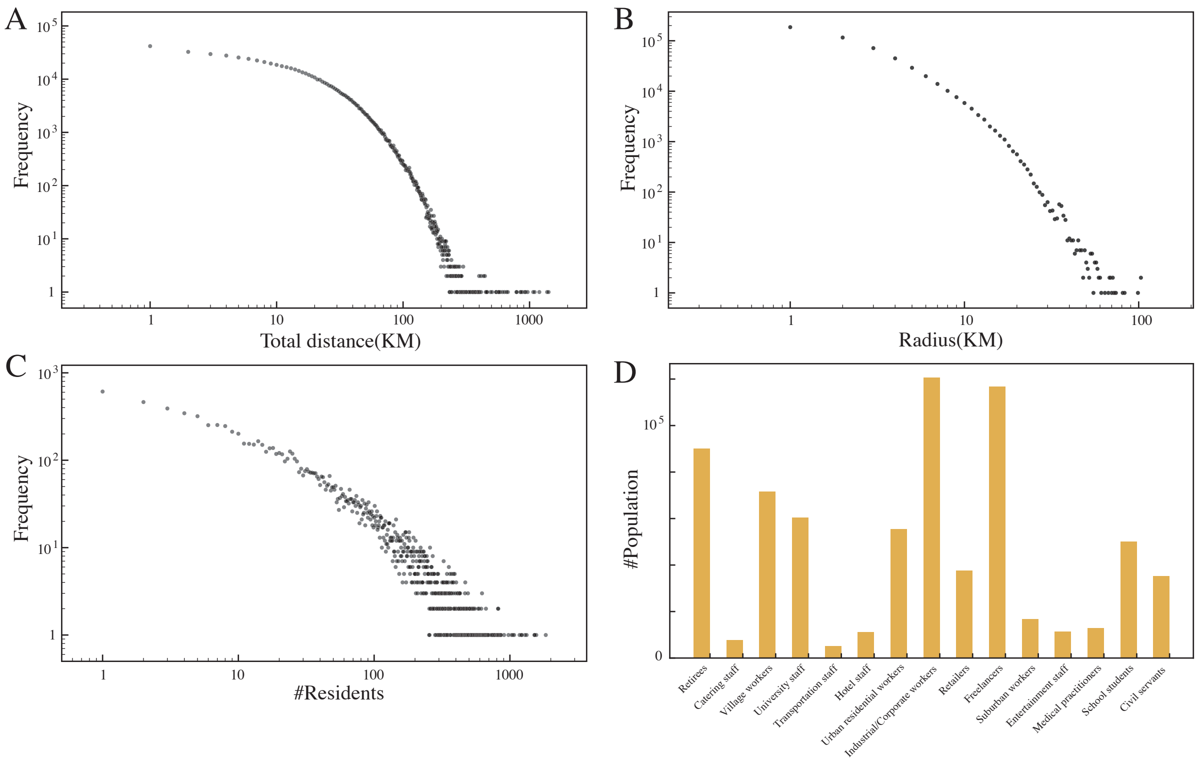

S1 Fig. The basic statistics of the data. (A) The distribution of traveling distance in the data. (B) The distribution of the radius of gyration of users in the data. The distribution follows a truncated power-law, indicating that most individuals move in a small area while a small number of individuals tend to travel a long distance. (C) The distribution of resident population of locations (corresponding to the area each cell-tower covers). The distribution follows a power-law form, suggesting that most locations are visited by few people while a few locations are very crowded. (D) The distribution of population in different professions. The three professions with the most population are industrial and corporate workers, freelancers, retirees.
