# Supplementary material for: A high-frequency mobility big-data reveals how COVID-19 spread across professions, locations and age groups
Source: PLoS Comput Biol. 2023 Apr 27;19(4):e1011083. doi: 10.1371/journal.pcbi.1011083 (PMC10168568; doi:10.1371/journal.pcbi.1011083)
Supplement: S2 Fig — (PDF) [file pcbi.1011083.s002.pdf]

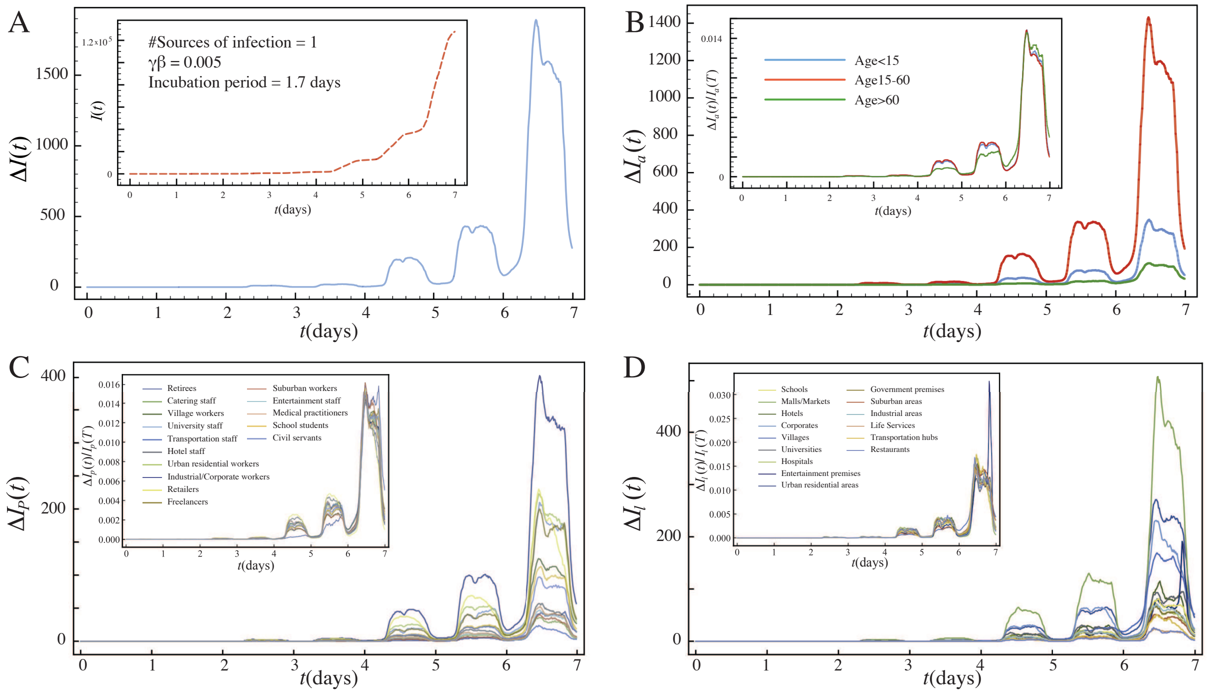

S2 Fig. The effect of the number of initial spreaders on the prevalence of the virus. Here, we set only 1 initial spreader, in contrast to the 70 initial spreaders in the main text of the paper. In order to avoid the spreading quickly dies out, we use a relatively larger infection rate as 0.005 (i.e. 2.5 times larger than the infection rate used in the paper). The rest of the parameters are the same as those used in the paper. (A) Given 1 initial spreader randomly located in the city, the evolution of the size of the infected population per quarter in the city. The inset shows the accumulated infected population in different days. (B) The evolution of the number of infected individuals (per quarter) in different age groups. Inset shows the evolution of the fraction of infected individuals (per quarter) in different age groups. (C) The evolution of the number of infected individuals (per quarter) of different professions in the city. The inset is the evolution of the fraction of infected individuals in different professions. (D) The evolution of the number of infected individuals in different location categories. The inset is the evolution of the fraction of infected individuals in different location categories.
