# Supplementary material for: A high-frequency mobility big-data reveals how COVID-19 spread across professions, locations and age groups
Source: PLoS Comput Biol. 2023 Apr 27;19(4):e1011083. doi: 10.1371/journal.pcbi.1011083 (PMC10168568; doi:10.1371/journal.pcbi.1011083)
Supplement: S4 Fig — (PDF) [file pcbi.1011083.s004.pdf]

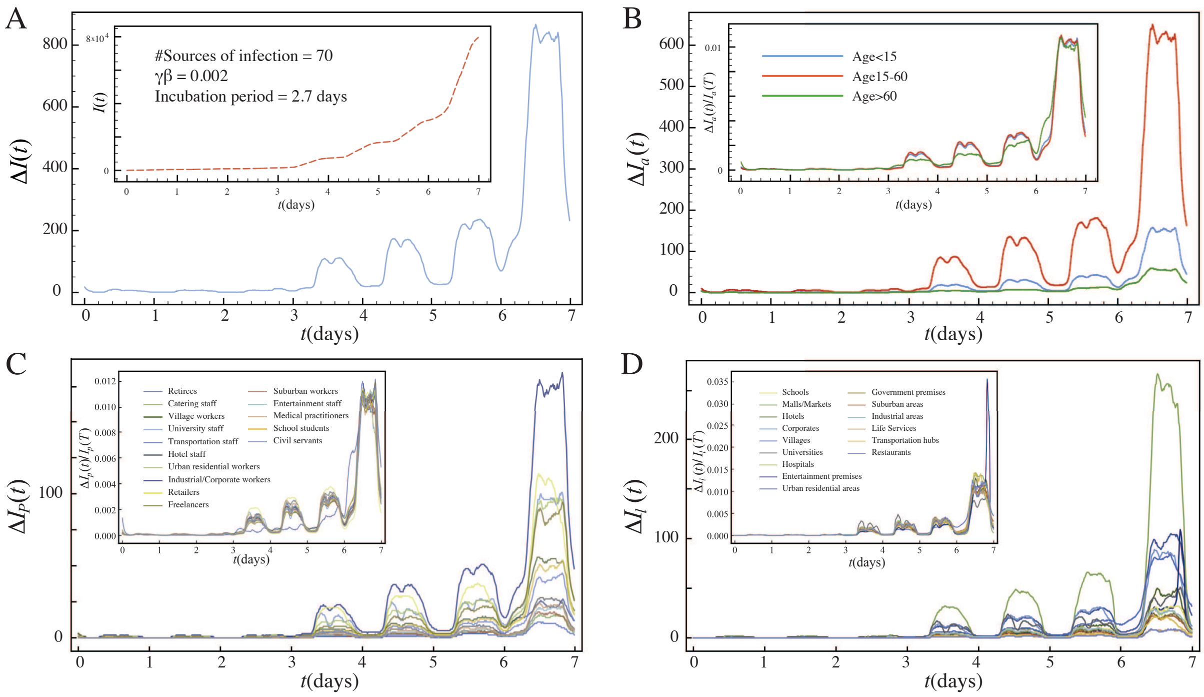

S4 Fig. The effect of a longer incubation period on the prevalence of the virus (an infected individual cannot infect others during the incubation period). Here, the incubation period is set as 2.7 days, smaller than the value 1.7 used in the main text of the paper. The rest parameters are the same as those used in the paper. (A) Given 70 initial spreaders randomly located in the city, the evolution of the number of infected population per quarter in the city. The inset shows the accumulated infected population in different days. (B) The evolution of the number of infected people (per quarter) of different ages. Inset shows the evolution of the fraction of infected people (per quarter) of different ages. (C) The evolution of the number of infected people (per quarter) of different professions in the city. The inset is the evolution of the fraction of infected people of different professions. (D) The evolution of the number of infected people in different types of locations. The inset is the evolution of the fraction of infected people in different types of locations.
