# Supplementary material for: A high-frequency mobility big-data reveals how COVID-19 spread across professions, locations and age groups
Source: PLoS Comput Biol. 2023 Apr 27;19(4):e1011083. doi: 10.1371/journal.pcbi.1011083 (PMC10168568; doi:10.1371/journal.pcbi.1011083)
Supplement: S5 Fig — (PDF) [file pcbi.1011083.s005.pdf]

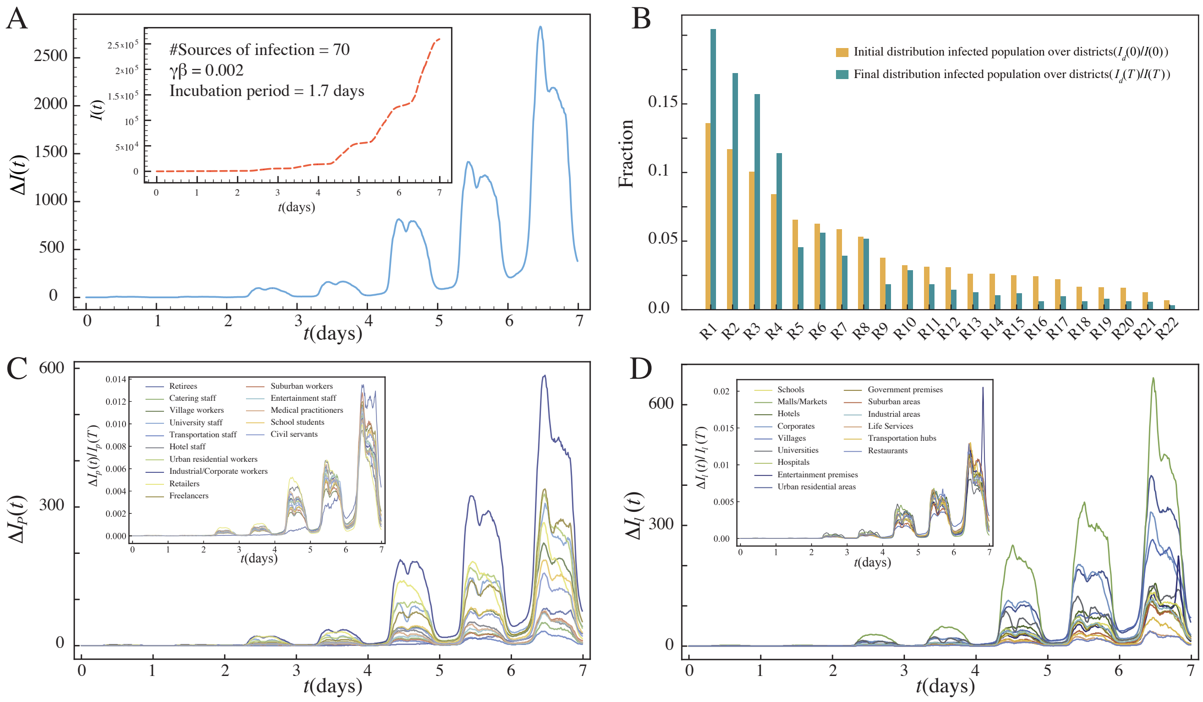

S5 Fig. The role of home infection rate on the prevalence of the virus. In this figure, we reduce the home infection rate to $\beta = 0.002$ which is the same as the infection rate outside. The rest parameters are the same as those used in the paper. (A) Given 70 initial spreaders randomly located in the city, the evolution of the number of infected population per quarter in the city. A significant periodic infection cycle can be observed, which is caused by the periodic human mobility patterns. The inset shows the accumulated infected population, i.e. $\Delta I(t)$, in different days. (B) The initial and the final distribution of initial individuals in different districts of the city. The districts are shown in the map in Fig 2 in the main text of the paper. (C) The evolution of the number of infected people (per quarter) of different professions in the city. The inset is the evolution of the fraction of infected people of different professions. (D) The evolution of the number of infected people in different types of locations. The inset is the evolution of the fraction of infected people in different types of locations.
