# Supplementary material for: A high-frequency mobility big-data reveals how COVID-19 spread across professions, locations and age groups
Source: PLoS Comput Biol. 2023 Apr 27;19(4):e1011083. doi: 10.1371/journal.pcbi.1011083 (PMC10168568; doi:10.1371/journal.pcbi.1011083)
Supplement: S6 Fig — (PDF) [file pcbi.1011083.s006.pdf]

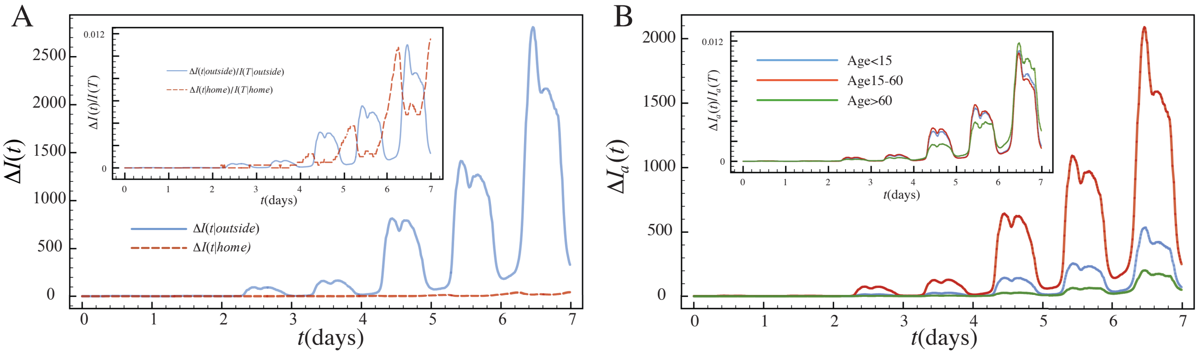

S6 Fig. The role of home infection rate on the prevalence of virus. In this figure, we reduce the home infection rate to $\beta = 0.002$ which is the same as the infection rate outside. The rest of the parameters are the same as those used in the paper. (A) The evolution of the number of individuals (per quarter) infected at home and the number of individuals (per quarter) infected outside. The inset shows the evolution of the fraction of infected individuals who are infected at home and outside, respectively. (B) The evolution of the number of infected individuals (per quarter) in different age groups. Inset shows the evolution of the fraction of infected individuals (per quarter) in different age groups.
