# Supplementary material for: A high-frequency mobility big-data reveals how COVID-19 spread across professions, locations and age groups
Source: PLoS Comput Biol. 2023 Apr 27;19(4):e1011083. doi: 10.1371/journal.pcbi.1011083 (PMC10168568; doi:10.1371/journal.pcbi.1011083)
Supplement: S7 Fig — (PDF) [file pcbi.1011083.s007.pdf]

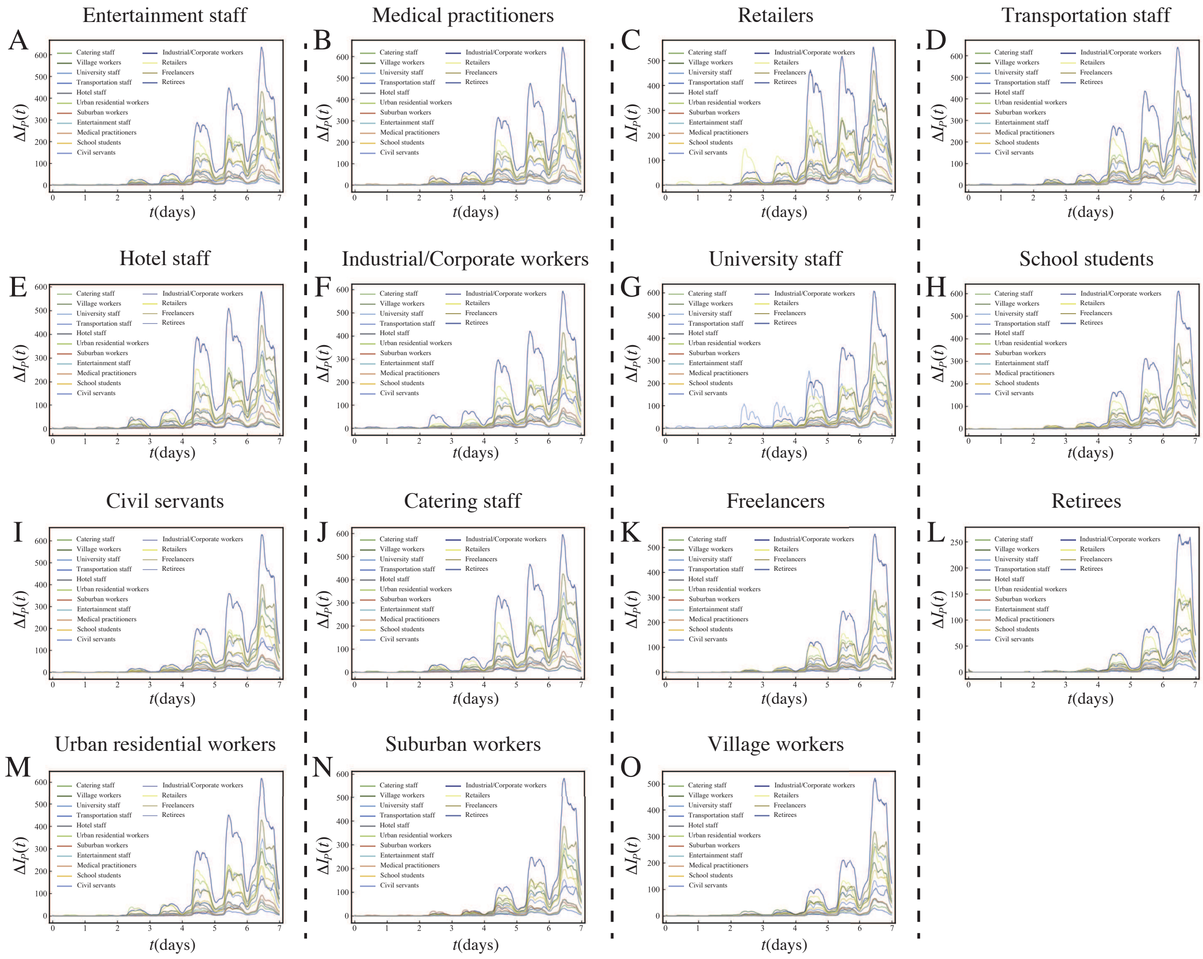

S7 Fig. We simulate the spreading results given that the infection starts at individuals of different professions, respectively. 70 initial spreaders are randomly selected from the population of a given profession. The spreading is continued with the intimate contact mechanism for 7 days. All parameters are the same as those used in the paper. The professions are (A) Entertainment staff, (B) Medical practitioner, (C) Retailer, (D) Transportation staff, (E) Hotel staff, (F) Industrial and Corporate Workers, (G) University staff, (H) School student, (I) Civil servant, (J) Catering staff, (K) Freelancer, (L) Retired, (M) Resident, (N) Rural resident, (O) Village resident. In all cases, the significant periodic infection cycle can be observed. However, the spreading initialized from different professions exhibits significant heterogeneity.
