# Supplementary material for: A high-frequency mobility big-data reveals how COVID-19 spread across professions, locations and age groups
Source: PLoS Comput Biol. 2023 Apr 27;19(4):e1011083. doi: 10.1371/journal.pcbi.1011083 (PMC10168568; doi:10.1371/journal.pcbi.1011083)
Supplement: S8 Fig — (PDF) [file pcbi.1011083.s008.pdf]

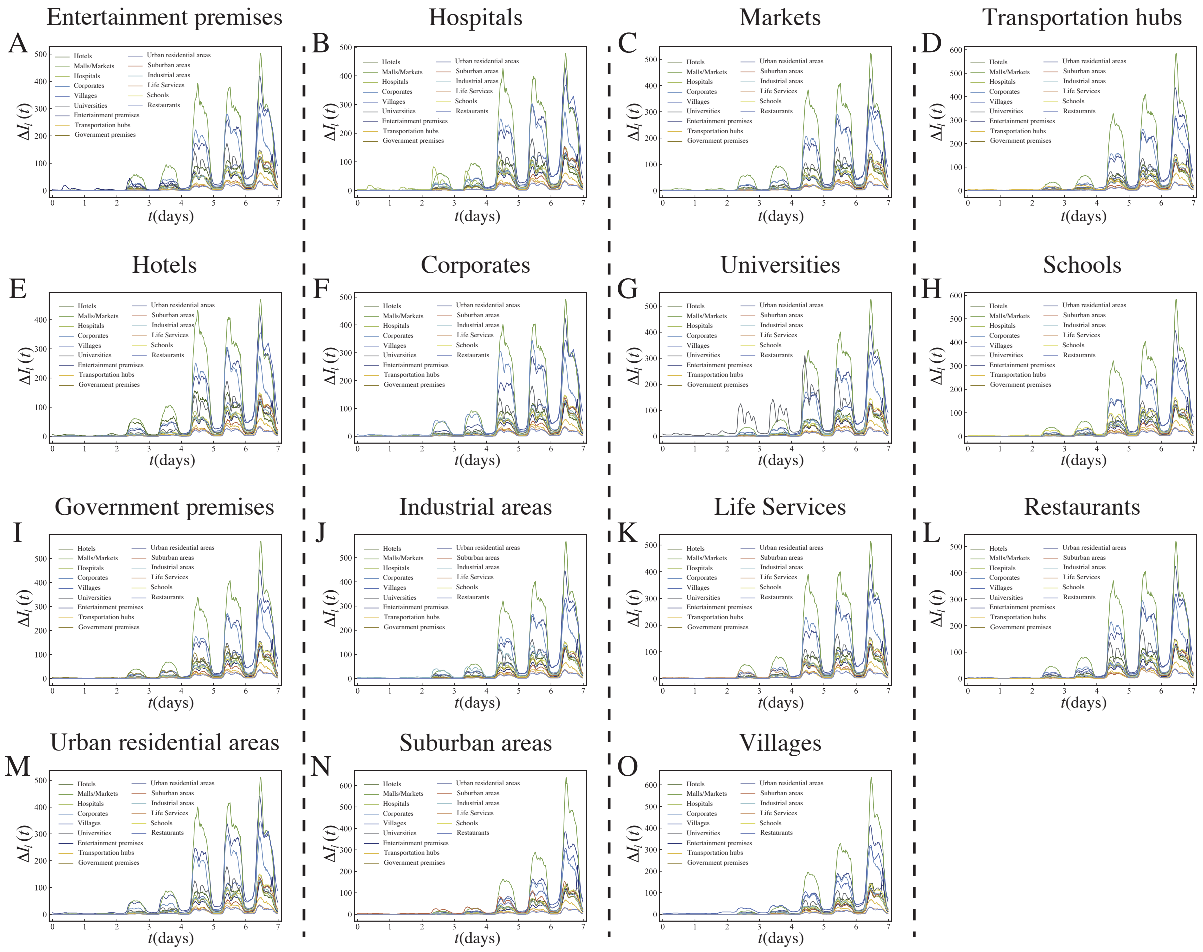

S8 Fig. We simulate the spreading results given that the infection starts at locations in different location categories, respectively. The initial spreaders are generated by the environmental infection. Assuming each individual would get infected with an infection rate 0.002 when visiting the designated location, the environmental infection is terminated when the number of initial spreaders reach 70. The spreading is continued with the intimate contact mechanism for 7 days. All parameters are the same as those used in the paper. The locations designated for environmental infection are (A) Entertainment premises, (B) Hospital, (C) Market, (D) Transportation hub, (E) Hotel, (F) Corporate, (G) University, (H) School, (I) Government premises, (J) Industrial area, (K) Life Services, (L) Restaurant, (M) Urban Residential area, (N) Suburban area. (O) Village. In all cases, the significant periodic infection cycle can be observed. However, the spreading initialized from different locations exhibits significant heterogeneity. In particular, the spreading evolution initialized from suburban areas or villages is different from that started in other locations, possibly due to the different human mobility patterns in urban and suburban area.
