# Supplementary material for: A high-frequency mobility big-data reveals how COVID-19 spread across professions, locations and age groups
Source: PLoS Comput Biol. 2023 Apr 27;19(4):e1011083. doi: 10.1371/journal.pcbi.1011083 (PMC10168568; doi:10.1371/journal.pcbi.1011083)
Supplement: S9 Fig — (PDF) [file pcbi.1011083.s009.pdf]

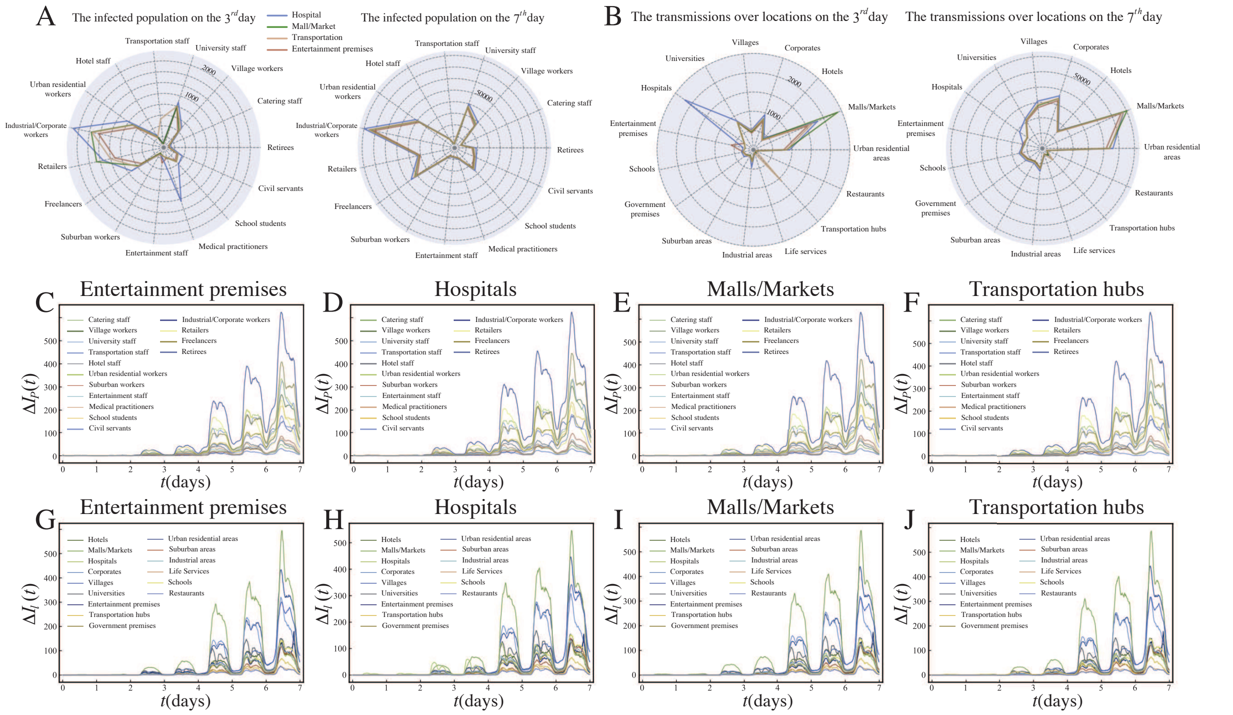

S9 Fig. The simulation results where transmissions through both contact and environment are considered. In this case, we first randomly select 70 individuals as the initial infected group. We then consider four different types of locations for environmental spreading, respectively. If a location is a source of environmental spreading, any individual who visits it would have a probability 0.002 to be infected. We consider respectively four different types of locations as the source, namely entertainment, hospital, market and transportation hub. For the location category "entertainment premises", we select 12 major locations labeled by this location category. For transportation type of locations, we select 20 major locations marked by this location category (mostly train stations, bus stations and airports). For hospital type of locations, we select 31 major locations labeled by this location category (mostly hospitals and institutions of disease control. For location category "markets", we select 17 major locations marked by this location catergory (mostly supermarkets). (A) The radar map showing the distribution of infection across professions until the third day and the last day. (B) The radar map showing the distribution of infected locations until the third day and the last day. (C-F) The evolution of the number of infected population of different professions per quarter in the city. (G-J) The evolution of the number of infected population in different locations per quarter in the city.
