# Supplementary material for: A high-frequency mobility big-data reveals how COVID-19 spread across professions, locations and age groups
Source: PLoS Comput Biol. 2023 Apr 27;19(4):e1011083. doi: 10.1371/journal.pcbi.1011083 (PMC10168568; doi:10.1371/journal.pcbi.1011083)
Supplement: S10 Fig — The figure shows the distribution of highest Pearson correlation in each year. (PDF) [file pcbi.1011083.s010.pdf]

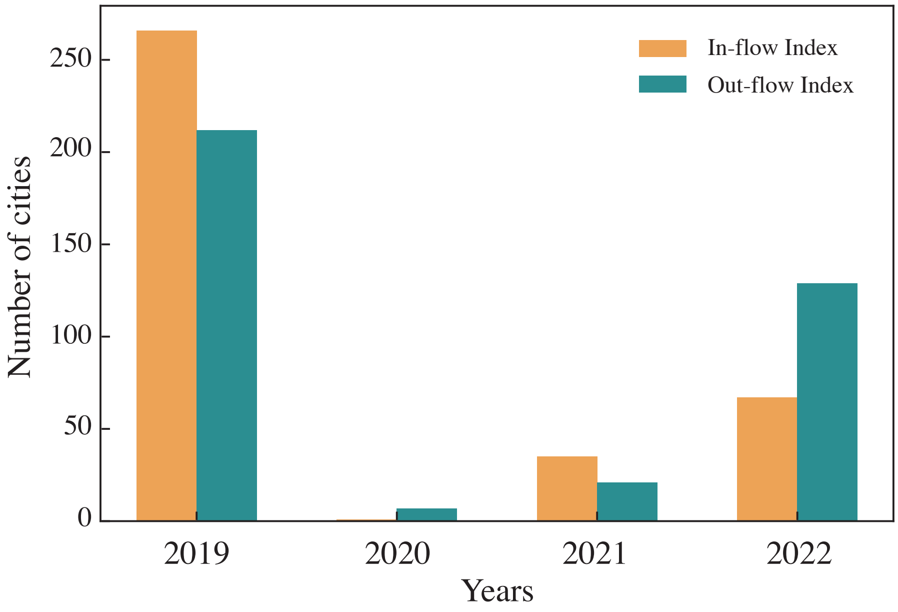

S10 Fig. We first calculate the Pearson correlation between the mobility patterns of each city in year 2019, 2020, 2021, 2022 and the patterns after opening up in year 2023. The figure shows the distribution of highest Pearson correlation in each year. The yellow bar represents in-flow mobility and the green bar represents out-flow mobility. The results show that the 2019 mobility data of most cities are closest to the 2023 mobility data.
