# Supplementary material for: A high-frequency mobility big-data reveals how COVID-19 spread across professions, locations and age groups
Source: PLoS Comput Biol. 2023 Apr 27;19(4):e1011083. doi: 10.1371/journal.pcbi.1011083 (PMC10168568; doi:10.1371/journal.pcbi.1011083)
Supplement: S1 Table — (PDF) [file pcbi.1011083.s011.pdf]

S1 Table. The population in different districts of Shijiazhuang city, together with the percentage of population in different age groups in each district.

District	Name	Population	Age $\in [0, 14]$	Age $\in [15, 60]$	Age beyond 60
R	Shijiazhuang (whole city)	10,640,458	19.5%	62.5%	18.0%
R1	Yuhua district	771,255	17.1%	69.1%	13.8%
R2	Qiaoxi district	979,646	15.9%	68.6%	15.5%
R3	Changan district	1,059,572	17.6%	67.2%	15.2%
R4	Xinhua district	802,057	17.0%	68.0%	15.0%
R5	Gaocheng district	741,068	21.0%	59.5%	19.5%
R6	Zhengding district	549,321	19.9%	62.3%	17.8%
R7	Xinji district	594,628	16.1%	57.8%	26.1%
R8	Luquan district	588,279	19.4%	63.6%	17.0%
R9	Jinzhou district	507,959	20.5%	57.2%	22.3%
R10	Luancheng district	378,689	19.9%	63.2%	16.9%
R11	Xinle district	478,529	22.5%	60.4%	17.1%
R12	Wuji district	451,377	21.4%	57.6%	21.0%
R13	Pingshan district	423,333	21.1%	56.5%	22.4%
R14	Zhao district	505,366	21.5%	57.9%	20.6%
R15	Yuanshi district	392,710	21.0%	60.0%	19.0%
R16	Xingtang district	376,627	22.3%	56.1%	21.6%
R17	Lingshou district	309,121	20.3%	59.2%	20.5%
R18	Zanhuang district	242,549	27.0%	54.7%	18.3%
R19	Jingxing district	250,989	16.7%	57.7%	25.6%
R20	Shenze district	215,806	18.4%	55.9%	25.7%
R21	Gaoyi district	178,368	23.4%	55.8%	20.8%
R22	Jingxingkuang district	77,015	15.8%	61.0%	23.2%
